# Evaluating antimicrobial stewardship strategies in candidemia: a novel desirability of outcome ranking (DOOR) analysis comparing blood culture versus T2Candida diagnostic approaches

**DOI:** 10.1128/jcm.00043-25

**Published:** 2025-04-11

**Authors:** Kaylee E. Caniff, Mohammed Al Musawa, Chloe Judd, Macy Shupp, Michael P. Veve, George Alangaden, Kimberly C. Claeys, Marco R. Scipione, Thomas J. Walsh, Michael J. Rybak

**Affiliations:** 1 Wayne State University Eugene Applebaum College of Pharmacy and Health Sciences 15538, Detroit, Michigan, USA; 2 Henry Ford Health System 2971, Detroit, Michigan, USA; 3 University of Maryland School of Pharmacy 15513, Baltimore, Maryland, USA; 4 Department of Pharmacy Services, Detroit Receiving Hospital, Detroit Medical Center 22944, Detroit, Michigan, USA; 5 Center for Innovative Therapeutics and Diagnostics, Richmond, Virginia, USA; 6 University of Maryland School of Medicine 12264, Baltimore, Maryland, USA; 7 Wayne State University School of Medicine 12267, Detroit, Michigan, USA; University of Calgary, Calgary, Alberta, Canada

**Keywords:** candidemia, invasive candidiasis, T2Candida, antimicrobial stewardship, rapid diagnostics

## Abstract

The T2Candida Panel (T2 Biosystems, Lexington, MA) is a rapid diagnostic test that detects *Candida* from whole blood within 3–5 hours. We developed and applied a desirability of outcome ranking (DOOR) analysis to investigate if an antimicrobial stewardship program (ASP) strategy centered on T2Candida diagnosis is associated with improved outcomes compared to an ASP strategy that relies on conventional blood culture diagnosis in critically ill patients with candidemia. This is a retrospective, observational cohort of patients with candidemia identified ≤72 h of intensive care unit admission at two medical centers in Detroit, MI (one T2Candida site and one blood culture site) from 2016 to 2023. Management strategies for candidemia were compared using an original DOOR analysis with inverse probability of treatment weighting (IPTW) to account for confounding. Two hundred patients were included, 100 from each site. Baseline illness severity, race, and *Candida* species varied between groups; however, source control procedures, echocardiogram, and ophthalmologic exam occurred at similar frequencies. T2Candida/ASP was associated with faster median (interquartile range [IQR]) detection of candidemia (7.0 [5.0–10.75] h vs 45.5 h [34.25–68.75], *P* < 0.001) and timelier median (IQR) initiation of directed antifungal therapy (6.0 [0–11.0] h vs 49.0 [34.0–77.0] h, *P* < 0.001). T2Candida/ASP patients had a 58.0% probability of achieving an overall better outcome compared to those managed with blood culture/ASP (95% confidence interval: 50.4–65.2%) in IPTW-adjusted DOOR analysis. An ASP strategy incorporating T2Candida was associated with an overall better patient outcome compared to patients managed via conventional blood culture diagnosis.

**IMPORTANCE:**

*Candida* species are a significant cause of bloodstream infections in critically ill patients. Conventional diagnostic methods, such as blood cultures, have poor sensitivity and delayed results. The T2Candida Panel is a diagnostic tool that rapidly detects *Candida* directly from the blood in 3–5 h, enabling faster initiation of antifungal therapy. Antimicrobial stewardship programs (ASPs) optimize the management of bloodstream infections and may benefit from incorporating T2Candida to improve patient outcomes. This study examined whether an ASP intervention based on T2Candida diagnosis, compared to one relying on traditional blood culture methods, could improve outcomes in candidemia using a desirability of outcome ranking (DOOR) analysis. The DOOR method provides a comprehensive evaluation by integrating multiple outcomes into a single end point, which is ideal given the complexity of patients with candidemia. The T2Candida/ASP intervention resulted in an overall better patient outcome, considering infectious complications, treatment failure, and all-cause mortality.

## INTRODUCTION

*Candida* species are an important cause of nosocomial bloodstream infections, particularly among critically ill patients (1–3). In the intensive care unit (ICU), candidemia occurs at least 5–10 times more frequently than in non-ICU wards, potentiated by frequent pathological and iatrogenic disruptions in immunologic function and the host microbiome (2, 4). Mortality rates associated with candidemia in the ICU exceed 40%, despite the advent of modern antifungal therapies (4). Moreover, the development of candidemia has been linked to increased ICU length of stay and substantial healthcare costs (5, 6).

A key challenge in the management of candidemia is the timely identification of infection using conventional methods. Some data suggest blood cultures are approximately 50% sensitive for detecting invasive disease (7–9). If *Candida* is successfully recovered, blood cultures are further hampered by a median time to positivity of 2–3 days (8). The use of non-culture-based methods, such as (1→3)-β-d-glucan or anti-mannan antigen assays, is also limited by a lack of robust sensitivity and specificity, particularly among ICU patients (8, 10). The empirical use of antifungal therapy in high-risk patients could overcome the limitations of these diagnostic tools; however, judicious use of these agents is required to limit unnecessary exposure that drives resistance and contributes to adverse drug effects (11–14). Although early initiation of antifungal therapy in patients with documented candidemia improves survival, the use of empirical antifungal therapy in high-risk non-neutropenic patients without culture-proven candidiasis has not demonstrated improved outcomes (15–19).

The T2Candida Panel (T2Candida; T2 Biosystems, Lexington, MA) is the first approved direct-from-blood *Candida* species detection assay in the United States. This diagnostic tool utilizes T2 magnetic resonance (T2MR) technology to detect the five *Candida* species that are responsible for greater than 90% of invasive disease within 3–5 h (9, 20). In its pivotal clinical trial, T2Candida exhibited an overall specificity of 99.4% and sensitivity of 91.1%, with a negative predictive value of at least 99% (20). Implementation of T2Candida in real-world practice has demonstrated a significant reduction in time to appropriate therapy (21–23).

Antimicrobial stewardship programs (ASPs) targeting candidemia have demonstrated improvements in achieving quality-of-care diagnostic procedures and interventions. Nevertheless, ASP strategies have not translated to improved patient outcomes, particularly when reliant upon blood culture diagnosis (24–27). T2Candida could further enhance the clinical benefits of ASP given its ability to rapidly detect candidemia, which could facilitate more timely intervention. However, there is a paucity of data comparing ASP strategies in patients with confirmed candidemia diagnosed via T2Candida compared to strategies dependent upon conventional blood cultures. We hypothesized that an ASP policy fundamentally based on T2Candida would improve the outcome of patients suffering from candidemia.

Given the complexity of critically ill patients with candidemia, assessing clinical outcomes independently as binary end points with traditional statistical analyses may fail to adequately capture the overall patient health status. To address this challenge, we developed and applied a desirability of outcome ranking (DOOR) analysis to globally assess the clinical outcomes of two management strategies in candidemia: T2Candida plus ASP compared to conventional blood culture diagnosis plus ASP. The DOOR methodology provides a more comprehensive evaluation of the overall benefits of an intervention by integrating multiple outcomes into a hierarchical end point (28, 29). By providing more detailed and nuanced evidence, this study aims to inform and advance the management of candidemia.

## MATERIALS AND METHODS

### Study design and patient population

This is a retrospective, observational cohort study conducted at the Detroit Medical Center (DMC) and Henry Ford Health (HFH) outcomes of patients with candidemia who were managed through ASP based on conventional blood culturing versus T2Candida. The DMC and HFH are large, academic healthcare systems serving the greater Detroit, Michigan area. Blood culture patients were selected from the DMC (blood culture/ASP group) and T2Candida patients were selected from HFH (T2Candida/ASP group). Patients ≥18 years were eligible for inclusion if they were diagnosed with candidemia, identified via positive T2Candida or blood culture, between 1 January 2016 and 31 December 2023, and were either in an ICU or admitted to an ICU within 72 h of the positive result. Patients were excluded if they met any of the following criteria: (i) receipt of antifungal therapy for a prophylaxis indication, (ii) were among a vulnerable group (prisoners, pregnant, or nursing patients), (iii) had a bacterial bloodstream co-infection, (iv) were COVID-19 positive at/during admission, or (v) died or were discharged to hospice care within 48 h of blood culture/T2Candida draw. Patients meeting inclusion and exclusion criteria were reviewed consecutively at each site until the targeted sample size was achieved.

Patient data were extracted from the electronic medical record manually and entered into the Research Electronic Data Capture (REDCap, Vanderbilt University) tool hosted by Wayne State University (30). Collected data elements included patient demographics, comorbidities, antimicrobial therapy information, relevant laboratory parameters and diagnostic tests, microbiologic data, infectious diseases (ID) consult information, pursuit of source control, and relevant clinical outcomes. This study was reviewed and approved by the Wayne State University, DMC, and HFH institutional review boards. A waiver of consent was received due to the retrospective design.

### ASP management strategies

At the DMC, antifungal therapy was initiated in response to positive blood cultures or empirically based upon the discretion of the primary medical team. Real-time alerts were sent to DMC ASP pharmacists in response to positive blood cultures with identified yeasts, who reviewed and acted upon the information to ensure the patient was initiated or continued on appropriate antifungal therapy. Conversely, at HFH, patients admitted to an ICU who continued to have fever and/or clinical worsening after at least 72 h of broad-spectrum antibiotics are initiated on echinocandin therapy, coupled with the attainment of T2Candida and blood cultures. Similar to the DMC, real-time alerts from T2Candida and blood culture results are sent to and enacted upon by ASP pharmacists. A negative T2Candida test prompted a reassessment of invasive candidiasis, potentially leading to the discontinuation of antifungal therapy if deemed appropriate. Follow-up T2Candida testing was discouraged if the purpose was to confirm infection clearance.

### Laboratory procedures

At the DMC, blood cultures were incubated in the BD BACTEC automated blood culture system (BD Diagnostic Systems, Franklin Lakes, NJ). All positive blood cultures underwent Gram staining and rapid diagnostic testing with Verigene gram-negative and gram-positive panels (Luminex Corporation, Austin, TX). Organism identification was performed with matrix-assisted laser desorption/ionization time of flight mass spectrometry. Routine antifungal susceptibility testing was performed on all *Candida* species isolated from blood cultures using gradient diffusion strips (bioMérieux, Durham, NC). At HFH, blood cultures were incubated in VersaTREK (TREK Diagnostic Systems, Cleveland, OH; January 2016 to January 2019) or BacT/Alert Virtuo BC System (bioMérieux, Durham, NC; February 2019 to December 2023). Positive blood cultures underwent Gram staining and testing with BioFire FilmArray Blood Culture Identification 2 (BCID2) Panel (bioMérieux). For yeast isolated from blood cultures, routine susceptibility was performed using Sensititre YeastOne (Thermo Fisher Scientific, Waltham, MA).

### Definitions

Severity of illness was quantified using the Acute Physiology and Chronic Health Evaluation II (APACHE II) score recording the worst physiologic parameters within 24 h of index blood culture or T2Candida (31). Baseline comorbidities were quantified with the Charlson Comorbidity Index (32). Severe sepsis was defined as having a sequential organ failure assessment score at the time of blood culture/T2Candida draw of ≥2, as this has previously been validated for incorporation into the Candida score (33). Candida species identified via blood culture or T2Candida were reported in categories as *C. albicans*/*C. tropicalis*, *C. krusei*/*C. glabrata*, or *C. parapsilosis* to remain consistent with T2Candida result output.

Empirical therapy was defined as the use of an antifungal agent prior to microbiological confirmation of candidemia via blood culture or T2Candida. Directed therapy was defined as treatment administered after blood culture/T2Candida result that was assumed to provide appropriate coverage of the identified organism based on available susceptibilities. However, patients with a positive T2Candida at HFH may not have had a corresponding positive blood culture for susceptibility testing. Thus, the use of fluconazole in these instances was assumed to be inappropriate if it was prescribed for a bloodstream infection caused by *C. krusei/C. glabrata* unless there was a culture with susceptibilities supporting fluconazole selection. Outcome definitions are outlined in the statistical analysis section.

### Statistical analysis

The sample size was determined *a priori*. It was calculated that a sample size of 160 (80 per arm) was needed to provide 90% power using a 2.5% significance level for one-sided Wilcoxon Mann-Whitney *U*-test to detect a difference in higher DOOR probability, described below, of at least 15%. The target sample size was increased to 200 (100 per arm) to ensure statistical robustness.

Descriptive statistics were utilized to analyze the baseline demographics, illness severity, infection characteristics, clinical management, and clinical outcomes. Unadjusted comparisons were conducted for categorical variables using the chi-squared test or Fisher’s exact test (for *n* < 5). For continuous variables, Student’s *t*-test was utilized for normally distributed variables, and the Wilcoxon Mann-Whitney *U*-test was utilized for non-normally distributed variables. A *P* value of <0.05 was considered statistically significant for two-tailed tests. The descriptive, bivariable outcome, and unadjusted DOOR analyses were performed using SPSS Statistics version 29.0 (IBM Corp., Armonk, NY). The weight-adjusted DOOR analysis was completed using an openly accessible web-based application (34, 35).

### Clinical outcomes

**Fig 1:**
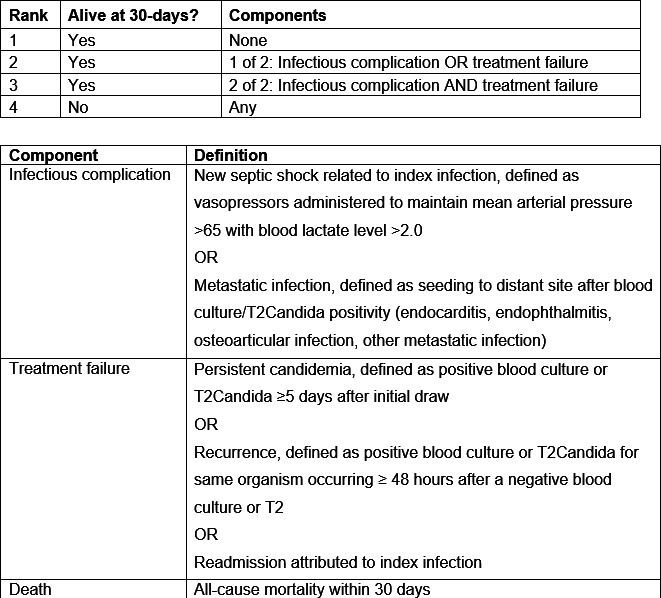
Desirability of outcome ranking (DOOR) analysis components.

The primary outcome was a DOOR analysis at 30 days following blood culture/T2Candida draw (Fig. 1). The DOOR outcome components were adapted from strategies that were previously developed for *Staphylococcus aureus* bloodstream infection and rapid diagnostic testing in gram-negative bloodstream infection (29, 36). The DOOR outcome in this study received review and revision from the study team, which was composed of ID researchers, physicians, and pharmacists, including those with specific expertise in candidemia.

In the DOOR analysis, patients were assigned a mutually exclusive hierarchical rank based on the overall status at 30 days post-blood culture or T2Candida draw. Rank 1 was the best possible outcome, indicating survival without the occurrence of either of the deleterious event components. Ranks 2 and 3 indicated survival but with the occurrence of one or two deleterious events, respectively. Rank 4 indicated the occurrence of all-cause mortality within the study period. Deleterious events were categorized as (i) treatment failure or (ii) infectious complications due to progression of infection (i.e., not initially present at the time of blood culture/T2Candida draw). After assignment of rank, the distribution of DOOR ranks was compared between groups with a Wilcoxon Mann-Whitney *U*-test. The DOOR probability, or the probability that a randomly selected case would have a better rank (more desirable outcome), was calculated. A DOOR probability of 50% indicates no difference between groups (37).

To adjust for the effect of selection bias between sites, an adjusted DOOR analysis was also performed after applying inverse probability of treatment weighting (IPTW) (38, 39). A non-parsimonious multivariable logistic regression model was developed to estimate the probability, or propensity score, of receiving T2Candida/ASP. Baseline demographic and infection characteristic variables with a *P* value of <0.1 and present in >10% in the univariate analysis were considered for entry into the model. The following characteristics were included in propensity score calculation: BMI, Black/African American race, APACHE II score, *C. albicans*/*C. tropicalis* species, and *C. krusei*/*C. glabrata* species. Empirical therapy administration, time to first therapy, time to directed therapy, and antifungal therapies administered were not considered for model entry as these variables were directly related to the ASP interventions and therefore on the causal pathway. Stabilized weights, based on the inverse of the propensity score, were applied to generate a pseudo-population. The prediction of the ability of the propensity score model was assessed with an area under the receiver operating characteristic (AU-ROC) curve. Standardized differences were examined to assess covariate balance before and after applying IPTW. A threshold of 10% was utilized to indicate a clinically meaningful imbalance. The DOOR analysis was then repeated using the pseudo-population generated from the application of IPTW.

Secondary outcomes included time to positive result from blood culture/T2Candida draw; first therapy administration, defined as time from blood culture/T2Candida draw to administration of first antifungal agent; time to directed therapy, defined as time from blood culture or T2Candida draw to administration of an antifungal agent with appropriate coverage, and individual components of the DOOR analysis (30-day mortality, treatment failure, persistent candidemia, occurrence of metastatic infection, and occurrence of septic shock).

## RESULTS

### Patient and infection characteristics

There were 200 patients included in the study: 100 patients diagnosed via blood culture (DMC patients) and 100 patients diagnosed via T2Candida (HFH patients). Baseline patient demographics and infection characteristics are described in Table 1. Overall, the median patient age was 62.0 (interquartile range [IQR]: 50.0–68.75) years and 53.5% of patients were male. T2Candida/ASP patients had a higher median BMI (28.25 [IQR: 23.0–34.5] vs 25.4 [IQR: 21.2–30.3], *P* = 0.004) and were more likely to be non-Hispanic Caucasian (57.0% vs 23.2%, *P* < 0.001), while blood culture/ASP patients were predominantly Black/African American (71.0% vs 32.0%, *P* < 0.001). Blood culture patients also exhibited a higher median APACHE II score at the time of index draw (28.5 [IQR: 20.0–30.0] vs 21.0 [IQR: 17.0–26.75], *P* < 0.001).

**TABLE 1:** Patient characteristics^*a*^

	Overall (*n* = 200)	Blood culture/ASP (*n* = 100)	T2Candida/ASP (*n* = 100)	*P* value
Age (years)	62.0 (50.0–68.75)	62.5 (52.25–69.75)	62.0 (48.25–68.0)	0.391
Male sex	107 (53.5)	54 (54.0)	53 (53.0)	0.887
BMI (kg/m^2^)	27.0 (22.2–31.9)	25.4 (21.2–30.3)	28.25 (23.0–34.5)	0.004
Race/ethnicity				
Black/African American	103 (51.5)	71 (71.0)	32 (32.0)	<0.001
Non-Hispanic Caucasian	80 (40.0)	23 (23.0)	57 (57.0)	<0.001
Other/unknown	17 (8.5)	6 (6.0)	11 (11.0)	0.213
Illness severity				
APACHE II score^*b*^	23.0 (18.0–30.0)	28.5 (20.0–33.0)	21.0 (17.0–26.75)	<0.001
Comorbidities				
Diabetes mellitus	68 (34.0)	33 (33.0)	35 (35.0)	0.765
HIV	5 (2.5)	3 (3.0)	2 (2.0)	0.651
Moderate-severe liver disease	14 (7.0)	4 (4.0)	10 (10.0)	0.164
Chronic dialysis	23 (11.5)	15 (15.0)	8 (8.0)	0.121
Tumor, no metastasis	9 (4.5)	3 (3.0)	6 (6.0)	0.498
Tumor with metastasis	13 (6.5)	10 (10.0)	3 (3.0)	0.082
Leukemia	2 (1.0)	0	2 (2.0)	0.497
Persons with injection drug use	21 (10.5)	14 (14.0)	7 (7.0)	0.106
Immunosuppressed^*c*^	42 (22.0)	25 (25.0)	17 (17.0)	0.165
Charlson Comorbidity Index	4.0 (3.0–6.0)	5.0 (3.0–7.0)	4.0 (2.25–6.0)	0.216
*Candida* risk factors				
Severe sepsis^*d*^	193 (96.5)	95 (95.0)	98 (98.0)	0.445
Surgery on ICU admission	55 (27.5)	24 (24.0)	31 (31.0)	0.268
Total parenteral nutrition	45 (22.5)	26 (26.0)	19 (19.0)	0.236
Multifocal *Candida* colonization	8 (4.0)	3 (3.0)	5 (5.0)	0.721
Candida score ≥3	82 (41.0)	39 (39.0)	43 (43.0)	0.565

^*a*^ Data are presented as number (%) and median (interquartile range [IQR]), as appropriate. BMI, body mass index; APACHE II, acute physiology and chronic health evaluation II; HIV, human immunodeficiency virus.

^*b*^ Calculated based on worst laboratory parameters within 24 h of blood culture or T2Candida draw.

^*c*^ Defined as absolute neutrophil count <500 cells/µL, CD4 <200 cells/mm^3^, and/or AIDS defining illness, splenectomy, solid organ transplant within the previous 90 days, bone marrow transplant within the previous 90 days, cytotoxic chemotherapy receipt in the previous 90 days, high-dose corticosteroids receipt (receipt of >40 mg of prednisone or equivalent for ≥2 weeks).

^*d*^ Sequential organ failure assessment score ≥2 at time blood culture or T2Candida draw (33).

In regard to infection characteristics and management (Table 2), *Candida* species distribution varied between groups, with T2Candida/ASP patients having a higher frequency of *C. albicans*/*C. tropicalis* (69.0% vs 26.0%, *P* < 0.001) and blood culture patients having a higher frequency of *C. krusei*/*C. glabrata* (44.0% vs 21.0%, *P* < 0.001). More than one *Candida* species was present in seven patients (3.5%). There were three patients in the blood culture/ASP group who possessed a *Candida* species not detectable by T2Candida. Nearly all patients from both groups received an ID consult, and patients were managed similarly with respect to the frequency of intravenous catheter removal, attainment of an echocardiogram, performance of ophthalmological examination, and receipt of a source control procedure. In accordance with the ASP procedure at HFH, empirical therapy was administered more frequently among the T2Candida/ASP group (48.0% vs 22.0%. *P* < 0.001). Most patients in both groups received an echinocandin as directed therapy, although more patients in the T2Candida/ASP group received fluconazole (55.0% vs 31.0%, *P* < 0.001), likely as step-down therapy following an initial echinocandin course and due to the higher frequency of *C. albicans*/*C. tropicalis*.

**TABLE 2:** Infection characteristics and management^*a*^

	Overall (*n* = 200)	Blood culture/ASP (*n* = 100)	T2Candida/ASP (*n* = 100)	*P* value
Organism				
*C. albicans*/*C. tropicalis*	105 (52.5)	36 (36.0)	69 (69.0)	<0.001
*C. krusei*/*C. glabrata*	65 (32.5)	44 (44.0)	21 (21.0)	<0.001
*C. parapsilosis*	33 (16.5)	18 (18.0)	15 (15.0)	0.568
Other	3 (1.5)	3 (1.5)	0	0.121
Polyfungemia	7 (3.5)	1 (1.0)	6 (6.0)	0.118
Infectious diseases consult	195 (97.5)	97 (97.0)	98 (98.0)	0.651
Therapeutic and diagnostic interventions				
Intravenous catheter removal	86 (43.0)	45 (45.0)	41 (41.0)	0.568
Echocardiogram	126 (63.0)	59 (59.0)	67 (67.0)	0.241
Ophthalmological examination	158 (79.0)	76 (76.0)	82 (82.0)	0.281
Source control procedure	90 (45.2)	46 (46.0)	44 (44.0)	0.727
Infection clearance confirmation via blood culture among blood culture positive patients	108/123 (87.8)	88 (88.0)	22/23 (95.7)	0.202
Negative follow-up blood culture or T2Candida within 120 h of index draw^*b*^	133 (66.5)	66 (66.0)	67 (67.0)	0.881
Empirical antifungal therapy administration^*c*^	70 (35.0)	22 (22.0)	48 (48.0)	<0.001
Echinocandin	56 (28.0)	13 (13.0)	43 (43.0)	<0.001
Fluconazole	14 (7.0)	9 (9.0)	5 (5.0)	0.268
Directed therapy^*d*^				
Echinocandin	172 (86.0)	89 (89.0)	83 (83.0)	0.221
Fluconazole	86 (43.0)	31 (31.0)	55 (55.0)	<0.001
Voriconazole	6 (3.0)	1 (1.0)	5 (5.0)	0.212
Isavuconazole	2 (1.0)	0	2 (2.0)	0.497
Flucytosine	1 (0.5)	1 (1.0)	0	1.000
Liposomal amphotericin	5 (2.5)	4 (4.0)	1 (1.0)	0.369
Amphotericin deoxycholate	1 (0.5)	0	1 (1.0)	1.000

^*a*^ Data are presented as number (%) and median (interquartile range [IQR]), as appropriate.

^*b*^ Among T2Candida/ASP patients, 66 had a negative blood culture and 1 had a negative T2Candida test within 120 h after the index T2Candida draw.

^*c*^ There were 14 patients who were receiving directed antifungal therapy for a prior culture-confirmed Candida infection at the time of blood culture or T2Candida result who were not considered to have empirical antifungal therapy.

^*d*^ Includes all directed therapies administered; *n* > 200 as patients could receive more than one agent as directed therapy.

### Clinical outcomes

Clinical outcomes are described in Table 3. T2Candida resulted at a median of 7.0 (IQR: 5.0–10.75) h following draw compared to 45.5 (34.25–68.75) h for blood culture results. T2Candida/ASP was associated with faster time to first antifungal therapy initiation (3.0 [IQR: 0–9.0] h vs 43.0 [IQR: 33.0–72.0] h, *P* < 0.001) and faster time to directed therapy (6.0 [IQR: 0–11.0] h vs 49.0 [IQR: 34.0–77.0] h, *P* < 0.001). Patients in the blood culture/ASP group experienced a greater frequency of infectious complications (49.0% vs 25.0%, *P* < 0.001) and treatment failure (13.0% vs 4.0%, *P* = 0.022).

**TABLE 3:** Clinical outcomes^*a*^

		Blood culture/ASP (*n* = 100)	T2Candida/ASP (*n* = 100)	*P* value
Time to positive result (h)^*b*^		45.5 (34.25–68.75)	7.0 (5.0–10.75)	<0.001
Time to first antifungal therapy (h)^*b*^		43.0 (33.0–72.0)	3.0 (0–9.0)	<0.001
Time to directed therapy (h)^*b*^		49.0 (34.0–77.0)	6.0 (0–11.0)	<0.001
Infectious complication		49 (49.0)	25 (25.0)	<0.001
Septic shock		37 (37.0)	17 (17.0)	0.001
Metastatic complication		22 (22.0)	11 (11.0)	0.036
Treatment failure		13 (13.0)	4 (4.0)	0.022
Persistent candidemia		9 (9.0)	2 (2.0)	0.030
30-day recurrence		1 (1.0)	1 (1.0)	1.000
Readmission due to index infection		3 (3.0)	1 (1.0)	0.621
30-day all-cause mortality		37 (37.0)	33 (33.0)	0.553

^*a*^ Data are presented as number (%) and median (interquartile range [IQR]), as appropriate. ICU, intensive care unit.

^*b*^ From blood culture or T2Candida draw.

**Fig 2:**
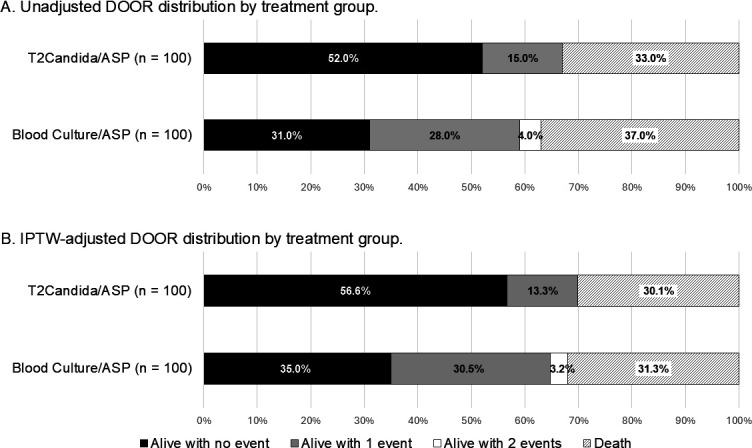
Unadjusted and adjusted desirability of outcome ranking (DOOR) distributions by treatment group.

Overall, 83 patients were alive without any deleterious events at 30 days. There were 43 patients who experienced one event and four who experienced two events. The unadjusted and adjusted DOOR distributions by treatment group are displayed in Fig. 2. The unadjusted probability of having a better outcome if randomly assigned to the T2Candida/ASP management approach was 58.3%, with a 95% confidence interval [CI] of 50.7–65.5% indicating statistical significance. After adjusting for confounders with IPTW, the DOOR probability remained significant (58.0%, 95% CI: 50.4–65.2%). The weighted standardized mean differences were <10% for investigated covariates (Table 4). The propensity score model had a good fit (Hosmer-Lemeshow statistic = 9.704, *P* = 0.286) and an AU-ROC curve demonstrated that it had an appropriate predictive index of 0.831.

**TABLE 4:** Standardized differences of variables used to generate the propensity score

	Standardized differences before applying IPTW (%)	Standardized differences after applying IPTW (%)	
BMI, mg/kg^2^	45.7	9.8	
Black/African American race	85.8	3.8	
APACHE II score	56.1	0.1	
*C. albicans*/*C. tropicalis* species	50.1	1.2	
*C. krusei*/*C. glabrata* species	79.9	5.0	

## DISCUSSION

Using DOOR analysis, an ASP intervention centered on T2Candida diagnosis resulted in an overall better clinical outcome compared to patients who were managed with an ASP strategy relying on blood culture diagnosis in this retrospective, observational cohort study. Consistent with other real-world studies, we found that T2Candida allowed for earlier identification of disease and faster time to appropriate antifungal therapy (21, 23, 40). Ultimately, prompt intervention likely led to the prevention of infectious complications and treatment failure resulting in an overall better clinical outcome among T2Candida/ASP patients. These findings are consistent with previous literature, which suggests that the use of rapid diagnostic tests in combination with ASP offers the greatest clinical benefit to patients with bloodstream infections, including those due to *Candida* species (41, 42).

It is important to interpret these results within the context of the full ASP intervention at HFH that was aimed at optimizing outcomes among patients with candidemia. T2Candida was the cornerstone of a bundled approach that included empirical echinocandin therapy, administered in approximately half of patients. Notably, the frequency of empirical therapy deviated from the HFH ASP policy, which recommended the administration of an echinocandin at the time of T2Candida draw in all patients. Clinicians may have deemed patients to be sufficiently stable to delay therapy until T2Candida results were available, a real-world practice pattern that demonstrates the utility of T2Candida. The high negative predictive value of this diagnostic tool supports rapid discontinuation of empiric therapy if the T2Candida test returns negative results. The poor sensitivity of blood cultures does not allow for them to be used to justify withholding or discontinuing empirical antifungal therapy in high-risk patients. Thus, T2Candida serves as an important ASP tool to optimize antifungal prescribing. Although not evaluated in the present study, previous analyses have found reduced antifungal use associated with the implementation of T2Candida (23, 43, 44).

This study was not powered to detect a difference in 30-day all-cause mortality; however, the T2Candida/ASP group showed no association with reduced mortality, despite their lower baseline APACHE II scores and faster initiation of therapy. Previous literature suggests that early initiation of antifungal therapy is associated with reduced mortality in patients with candidemia (16, 17). This paradigm may no longer strictly apply due to the increased comorbidity burden of critically ill patients, who may experience mortality from causes other than candidemia (45). Treatment practices have also evolved as echinocandins are now recommended as first-line agents in critically ill patients (9, 46). The majority of patients included in this study received directed treatment with an echinocandin agent, which could perhaps offer some survival benefit regardless of baseline illness severity or slight delays in treatment initiation (46, 47). Nevertheless, as illustrated by our findings, a reduction in time to therapy may confer other advantages, such as decreased infectious complications and treatment failure.

The use of the DOOR analysis in our study provided a more nuanced and detailed perspective of the overall clinical outcome of patients with candidemia. To our knowledge, no prior study has utilized a DOOR analysis to examine clinical outcomes associated with candidemia diagnosis or management. An exploratory DOOR analysis found that the use of the (1→3)-β-d-glucan assay to direct initiation of antifungal therapy was more cost-effective compared to a hypothetical initiation of empirical therapy in all high-risk patients (48). However, this analysis only assessed the cost-effectiveness of initiating or withholding antifungal therapy based on subsequent blood culture results and did not evaluate the actual clinical outcome of the patient. Our DOOR analysis provides comprehensive information regarding the clinical status of critically ill patients with candidemia that is not available with bivariate end points alone.

Although it is possible that false positives were included within our data set, we expect this to be minimally contributing to our findings. Blood cultures have limited sensitivity for *Candida* spp., ranging from 28% to 58% in patients with postmortem-confirmed invasive candidiasis, depending on the extent of deep tissue infection (7, 8). The detection of candidemia by T2MR technology depends upon the presence of an organism and does not detect circulating *Candida* DNA (DNA-emia), thus minimizing contamination-driven false positive specimens. In the original study of the T2MR system, it reproducibly detected five *Candida* species in whole blood, with a lower limit of detection of one colony-forming unit/milliliter. Additionally, it had a 98% positive agreement and 100% negative agreement in comparison to spiked blood samples (49). This was further confirmed in the clinical trial setting where T2Candida demonstrated 91.1% sensitivity compared to contrived specimens (20). Attesting to the greater sensitivity of T2Candida in comparison to blood cultures, several studies have demonstrated a higher level of sensitivity for detection in cases of intra-abdominal candidiasis with negative blood culture (50, 51). Other studies of T2Candida that have analyzed T2Candida positive/blood culture-negative cases with clinical adjudication have identified probable or possible invasive candidiasis in other sites (23, 52–54).

There are several limitations to this study. First, this was a retrospective analysis comparing candidemia ASP management strategies in two different healthcare systems. Although both are academic institutions serving an urban population within the metro Detroit area, there were significant baseline differences between groups. Moreover, the sample was not derived through random sampling given pragmatic limitations. We attempted to adjust for confounders utilizing IPTW, but there may be unmeasured covariates not accounted for in this adjustment. Second, there is currently no consensus DOOR definition for candidemia. We created the DOOR primary outcome for candidemia based on relevant end points modeled after DOOR components previously developed for bacterial bloodstream infections (29, 36). However, the DOOR scale utilized herein has not been widely validated or standardized. Our DOOR components were developed collaboratively with our study team, but ideally, the development of a DOOR outcome should include even broader input from patients and clinicians from various practice settings (37). Next, there is a particular challenge in identifying persistent candidemia in HFH patients via T2Candida, as the ASP protocol discouraged repeat T2Candida testing. However, a similar frequency of patients at each site had a negative blood culture drawn within 120 h of the index blood culture/T2Candida test. Thus, there was a similar opportunity for detection of this outcome in both groups. Finally, it is possible patients with an initial test with indeterminate results were included if T2Candida was re-run with a positive result. However, the number of such occurrences is likely to be small and is not expected to significantly impact the study findings.

In conclusion, we developed and applied a novel DOOR analysis to assess the clinical impact of rapid molecular diagnosis within ASP. We found that the use of T2Candida as part of an ASP intervention was associated with an overall better clinical outcome in patients with candidemia, primarily driven by a reduction in infectious complications. Future analyses should continue to refine DOOR methodology in evaluating rapid molecular diagnostics on the outcome of candidemia, as well as to compare T2Candida practice strategies to further optimize patient outcomes and healthcare costs.

## References

[1] Sydnor ERM, Perl TM. 2011. Hospital epidemiology and infection control in acute-care settings. Clin Microbiol Rev 24:141–173. doi:10.1128/CMR.00027-10 21233510 PMC3021207

[2] Hankovszky P, Társy D, Öveges N, Molnár Z. 2015. Invasive Candida infections in the ICU: diagnosis and therapy. J Crit Care Med 1:129–139. doi:10.1515/jccm-2015-0025 PMC5953294 29967821

[3] Tsay SV, Mu Y, Williams S, Epson E, Nadle J, Bamberg WM, Barter DM, Johnston HL, Farley MM, Harb S. 2020. Burden of candidemia in the United States, 2017. Clin Infect Dis 71:e449–e453. doi:10.1093/cid/ciaa193 32107534

[4] Logan C, Martin-Loeches I, Bicanic T. 2020. Invasive candidiasis in critical care: challenges and future directions. Intensive Care Med 46:2001–2014. doi:10.1007/s00134-020-06240-x 32990778

[5] Epelbaum O, Chasan R. 2017. Candidemia in the intensive care unit. Clin Chest Med 38:493–509. doi:10.1016/j.ccm.2017.04.010 28797491

[6] Bloos F, Bayer O, Sachse S, Straube E, Reinhart K, Kortgen A. 2013. Attributable costs of patients with candidemia and potential implications of polymerase chain reaction-based pathogen detection on antifungal therapy in patients with sepsis. J Crit Care 28:2–8. doi:10.1016/j.jcrc.2012.07.011 22999484

[7] Berenguer J, Buck M, Witebsky F, Stock F, Pizzo PA, Walsh TJ. 1993. Lysis-centrifugation blood cultures in the detection of tissue-proven invasive candidiasis. disseminated versus single-organ infection. Diagn Microbiol Infect Dis 17:103–109. doi:10.1016/0732-8893(93)90020-8 8243032

[8] Clancy CJ, Nguyen MH. 2013. Finding the “missing 50%” of invasive candidiasis: how nonculture diagnostics will improve understanding of disease spectrum and transform patient care. Clin Infect Dis 56:1284–1292. doi:10.1093/cid/cit006 23315320

[9] Pappas PG, Kauffman CA, Andes DR, Clancy CJ, Marr KA, Ostrosky-Zeichner L, Reboli AC, Schuster MG, Vazquez JA, Walsh TJ, Zaoutis TE, Sobel JD. 2016. Clinical practice guideline for the management of candidiasis: 2016 update by the infectious diseases society of America. Clin Infect Dis 62:e1–50. doi:10.1093/cid/civ933 26679628 PMC4725385

[10] Wheat LJ. 2009. Approach to the diagnosis of invasive aspergillosis and candidiasis. Clin Chest Med 30:367–377, doi:10.1016/j.ccm.2009.02.012 19375641

[11] Alexander BD, Johnson MD, Pfeiffer CD, Jiménez-Ortigosa C, Catania J, Booker R, Castanheira M, Messer SA, Perlin DS, Pfaller MA. 2013. Increasing echinocandin resistance in Candida glabrata: clinical failure correlates with presence of FKS mutations and elevated minimum inhibitory concentrations. Clin Infect Dis 56:1724–1732. doi:10.1093/cid/cit136 23487382 PMC3658363

[12] Garnacho-Montero J, Díaz-Martín A, García-Cabrera E, Ruiz Pérez de Pipaón M, Hernández-Caballero C, Aznar-Martín J, Cisneros JM, Ortiz-Leyba C. 2010. Risk factors for fluconazole-resistant candidemia. Antimicrob Agents Chemother 54:3149–3154. doi:10.1128/AAC.00479-10 20498325 PMC2916332

[13] Prasad PA, Fisher BT, Coffin SE, Walsh TJ, McGowan KL, Gross R, Zaoutis TE. 2013. Pediatric risk factors for candidemia secondary to Candida glabrata and Candida krusei species. J Pediatric Infect Dis Soc 2:263–266. doi:10.1093/jpids/pis093 24009984 PMC3761321

[14] Jacobs SE, Jacobs JL, Dennis EK, Taimur S, Rana M, Patel D, Gitman M, Patel G, Schaefer S, Iyer K, Moon J, Adams V, Lerner P, Walsh TJ, Zhu Y, Anower MR, Vaidya MM, Chaturvedi S, Chaturvedi V. 2022. Candida auris pan-drug-resistant to four classes of antifungal agents. Antimicrob Agents Chemother 66:e0005322. doi:10.1128/aac.00053-22 35770999 PMC9295560

[15] Morrell M, Fraser VJ, Kollef MH. 2005. Delaying the empiric treatment of Candida bloodstream infection until positive blood culture results are obtained: a potential risk factor for hospital mortality. Antimicrob Agents Chemother 49:3640–3645. doi:10.1128/AAC.49.9.3640-3645.2005 16127033 PMC1195428

[16] Garey KW, Rege M, Pai MP, Mingo DE, Suda KJ, Turpin RS, Bearden DT. 2006. Time to initiation of fluconazole therapy impacts mortality in patients with candidemia: a multi-institutional study. Clin Infect Dis 43:25–31. doi:10.1086/504810 16758414

[17] Patel GP, Simon D, Scheetz M, Crank CW, Lodise T, Patel N. 2009. The effect of time to antifungal therapy on mortality in candidemia associated septic shock. Am J Ther 16:508–511. doi:10.1097/MJT.0b013e3181a1afb7 19531934

[18] Schuster MG, Edwards JE, Sobel JD, Darouiche RO, Karchmer AW, Hadley S, Slotman G, Panzer H, Biswas P, Rex JH. 2008. Empirical fluconazole versus placebo for intensive care unit patients: a randomized trial. Ann Intern Med 149:83–90. doi:10.7326/0003-4819-149-2-200807150-00004 18626047

[19] Timsit JF, Azoulay E, Schwebel C, Charles PE, Cornet M, Souweine B, Klouche K, Jaber S, Trouillet JL, Bruneel F. 2016. Empirical micafungin treatment and survival without invasive fungal infection in adults with ICU-acquired sepsis, candida colonization, and multiple organ failure: the empiricus randomized clinical trial. JAMA 316:1555–1564. doi:10.1001/jama.2016.14655 27706483

[20] Mylonakis E, Clancy CJ, Ostrosky-Zeichner L, Garey KW, Alangaden GJ, Vazquez JA, Groeger JS, Judson MA, Vinagre Y-M, Heard SO, Zervou FN, Zacharioudakis IM, Kontoyiannis DP, Pappas PG. 2015. T2 magnetic resonance assay for the rapid diagnosis of candidemia in whole blood: a clinical trial. Clin Infect Dis 60:892–899. doi:10.1093/cid/ciu959 25586686

[21] Patch ME, Weisz E, Cubillos A, Estrada SJ, Pfaller MA. 2018. Impact of rapid, culture-independent diagnosis of candidaemia and invasive candidiasis in a community health system. J Antimicrob Chemother 73:iv27–iv30. doi:10.1093/jac/dky046 29608750

[22] Wilson GM, Suda KJ, Fitzpatrick MA, Bartle B, Pfeiffer CD, Jones M, Rubin MA, Perencevich E, Evans M, Evans CT. 2021. Risk factors associated with carbapenemase-producing carbapenem-resistant Enterobacteriaceae positive cultures in a cohort of US veterans. Clin Infect Dis 73:1370–1378. doi:10.1093/cid/ciab415 33973631

[23] O’Donnell M, Shields RK, Marini RV, Groetzinger LM, Potoski BA, Falcione BA, Shah S, McCreary EK, Clarke L, Brant E, McVerry BJ, Liegey S, Pasculle AW, Clancy CJ, Nguyen MH. 2023. Stewardship-guided T2Candida testing shortens time to antifungal treatment and reduces antifungal usage among medical intensive care unit patients with septic shock.. Open Forum Infect Dis 10:ofad538. doi:10.1093/ofid/ofad538 38023565 PMC10651185

[24] Antworth A, Collins CD, Kunapuli A. 2013. Impact of an antimicrobial stewardship program comprehensive care bundle on management of candidemia. Pharmacother J Hum Pharmacol Drug Ther 33:137–143. doi:10.1002/phar.1186 23355283

[25] Reed EE, West JE, Keating EA, Pancholi P, Balada-Llasat JM, Mangino JE, Bauer KA, Goff DA. 2014. Improving the management of candidemia through antimicrobial stewardship interventions. Diagn Microbiol Infect Dis 78:157–161. doi:10.1016/j.diagmicrobio.2013.11.012 24316015

[26] Pettit NN, Han Z, Nguyen CT, Choksi A, Charnot-Katsikas A, Beavis KG, Tesic V, Pisano J. 2019. Antimicrobial stewardship review of automated candidemia alerts using the epic stewardship module improves bundle-of-care adherence.. Open Forum Infect Dis 6:ofz412. doi:10.1093/ofid/ofz412 31660370 PMC6788339

[27] Ryder JH, Van Schooneveld TC, Lyden E, El Ramahi R, Stohs EJ. 2023. The interplay of infectious diseases consultation and antimicrobial stewardship in candidemia outcomes: a retrospective cohort study from 2016 to 2019. Infect Control Hosp Epidemiol 44:1102–1107. doi:10.1017/ice.2022.209 36082773

[28] Evans SR, Rubin D, Follmann D, Pennello G, Huskins WC, Powers JH, Schoenfeld D, Chuang-Stein C, Cosgrove SE, Fowler VG, Lautenbach E, Chambers HF. 2015. Desirability of outcome ranking (DOOR) and response adjusted for duration of antibiotic risk (RADAR). Clin Infect Dis 61:800–806. doi:10.1093/cid/civ495 26113652 PMC4542892

[29] Doernberg SB, Tran TTT, Tong SYC, Paul M, Yahav D, Davis JS, Leibovici L, Boucher HW, Corey GR, Cosgrove SE, Chambers HF, Fowler VG, Evans SR, Holland TL. 2019. Good studies evaluate the disease while great studies evaluate the patient: development and application of a desirability of outcome ranking endpoint for Staphylococcus aureus bloodstream infection. Clin Infect Dis 68:1691–1698. doi:10.1093/cid/ciy766 30321315 PMC6495020

[30] Harris PA, Taylor R, Thielke R, Payne J, Gonzalez N, Conde JG. 2009. Research electronic data capture (REDCap)-a metadata-driven methodology and workflow process for providing translational research informatics support. J Biomed Inform 42:377–381. doi:10.1016/j.jbi.2008.08.010 18929686 PMC2700030

[31] Knaus WA, Draper EA, Wagner DP, Zimmerman JE. 1985. APACHE II: a severity of disease classification system. Crit Care Med 13:818–829. 3928249

[32] Charlson ME, Pompei P, Ales KL, MacKenzie CR. 1987. A new method of classifying prognostic comorbidity in longitudinal studies: development and validation. J Chronic Dis 40:373–383. doi:10.1016/0021-9681(87)90171-8 3558716

[33] Li D, Zhang J, Han W, Bai G, Cheng W, Cui N. 2020. Evaluation of the updated “Candida score” with sepsis 3.0 criteria in critically ill patients. Ann Transl Med 8:917. doi:10.21037/atm-20-995 32953717 PMC7475415

[34] Antibiotic resistance leadership group. 2024. Interactive web-based app for analyzing desirability of outcome ranking (DOOR)*.* Available from: https://methods.bsc.gwu.edu/web/methods/door-professional-edition

[35] Hamasaki T, He Y, Evans SR. 2023. 369. The DOOR is open: a web-based application for analyzing the desirability of outcome ranking. Open Forum Infect Dis 10. doi:10.1093/ofid/ofad500.439

[36] Study details | fast antibiotic susceptibility testing for gram negative bacteremia trial | clinicaltrials.Gov. 2024. Available from: https://clinicaltrials.gov/study/NCT06174649

[37] Ong SWX, Petersiel N, Loewenthal MR, Daneman N, Tong SYC, Davis JS. 2023. Unlocking the DOOR-how to design, apply, analyse, and interpret desirability of outcome ranking endpoints in infectious diseases clinical trials. Clin Microbiol Infect 29:1024–1030. doi:10.1016/j.cmi.2023.05.003 37179006

[38] Austin PC, Stuart EA. 2015. Moving towards best practice when using inverse probability of treatment weighting (IPTW) using the propensity score to estimate causal treatment effects in observational studies. Stat Med 34:3661–3679. doi:10.1002/sim.6607 26238958 PMC4626409

[39] Chesnaye NC, Stel VS, Tripepi G, Dekker FW, Fu EL, Zoccali C, Jager KJ. 2022. An introduction to inverse probability of treatment weighting in observational research. Clin Kidney J 15:14–20. doi:10.1093/ckj/sfab158 35035932 PMC8757413

[40] Wilson NM, Alangaden G, Tibbetts RJ, Samuel LP, Davis SL, Kenney RM. 2018. T2 magnetic resonance assay improves timely management of candidemia. J Fungi (Basel) 4:45. doi:10.3390/jof4020045 29617284 PMC6023470

[41] Timbrook TT, Morton JB, McConeghy KW, Caffrey AR, Mylonakis E, LaPlante KL. 2017. The effect of molecular rapid diagnostic testing on clinical outcomes in bloodstream infections: a systematic review and meta-analysis.. Clin Infect Dis 64:15–23. doi:10.1093/cid/ciw649 27678085

[42] Banerjee R, Teng CB, Cunningham SA, Ihde SM, Steckelberg JM, Moriarty JP, Shah ND, Mandrekar JN, Patel R. 2015. Randomized trial of rapid multiplex polymerase chain reaction-based blood culture identification and susceptibility testing. Clin Infect Dis 61:1071–1080. doi:10.1093/cid/civ447 26197846 PMC4560903

[43] Gill CM, Kenney RM, Hencken L, Mlynarek ME, Alangaden GJ, Samuel LP, Davis SL. 2019. T2 Candida versus beta-D-glucan to facilitate antifungal discontinuation in the intensive care unit. Diagn Microbiol Infect Dis 95:162–165. doi:10.1016/j.diagmicrobio.2019.04.016 31248660

[44] Zacharioudakis IM, Zervou FN, Marsh K, Siegfried J, Yang J, Decano A, Dubrovskaya Y, Mazo D, Aguero-Rosenfeld M. 2024. Utility of incorporation of beta-D-glucan and T2Candida testing for diagnosis and treatment of candidemia. Diagn Microbiol Infect Dis 108:116107. doi:10.1016/j.diagmicrobio.2023.116107 38071859

[45] Lamoth F, Lockhart SR, Berkow EL, Calandra T. 2018. Changes in the epidemiological landscape of invasive candidiasis. J Antimicrob Chemother 73:i4–i13. doi:10.1093/jac/dkx444 29304207 PMC11931512

[46] Cornely FB, Cornely OA, Salmanton-García J, Koehler FC, Koehler P, Seifert H, Wingen-Heimann S, Mellinghoff SC. 2020. Attributable mortality of candidemia after introduction of echinocandins. Mycoses 63:1373–1381. doi:10.1111/myc.13177 32885534

[47] Reboli AC, Rotstein C, Pappas PG, Chapman SW, Kett DH, Kumar D, Betts R, Wible M, Goldstein BP, Schranz J, Krause DS, Walsh TJ, Anidulafungin Study Group. 2007. Anidulafungin versus fluconazole for invasive candidiasis. N Engl J Med 356:2472–2482. doi:10.1056/NEJMoa066906 17568028

[48] Giacobbe DR, Signori A, Tumbarello M, Ungaro R, Sarteschi G, Furfaro E, Mikulska M, Sanguinetti M, Posteraro B, Losito AR, De Pascale G, Del Bono V, Viscoli C. 2019. Desirability of outcome ranking (DOOR) for comparing diagnostic tools and early therapeutic choices in patients with suspected candidemia. Eur J Clin Microbiol Infect Dis 38:413–417. doi:10.1007/s10096-018-3441-1 30506332

[49] Neely LA, Audeh M, Phung NA, Min M, Suchocki A, Plourde D, Blanco M, Demas V, Skewis LR, Anagnostou T, Coleman JJ, Wellman P, Mylonakis E, Lowery TJ. 2013. T2 magnetic resonance enables nanoparticle-mediated rapid detection of candidemia in whole blood. Sci Transl Med 5:182ra54. doi:10.1126/scitranslmed.3005377 23616121

[50] Arendrup MC, Andersen JS, Holten MK, Krarup KB, Reiter N, Schierbeck J, Helleberg M. 2019. Diagnostic performance of T2Candida among ICU patients with risk factors for invasive candidiasis. Open Forum Infect Dis 6:ofz136. doi:10.1093/ofid/ofz136 31069244 PMC6501878

[51] Lamoth F, Clancy CJ, Tissot F, Squires K, Eggimann P, Flückiger U, Siegemund M, Orasch C, Zimmerli S, Calandra T, Marchetti O, Nguyen MH, Bochud PY. 2020. Performance of the T2 Candida panel for the diagnosis of intra-abdominal candidiasis. Open Forum Infect Dis 7:ofaa075. doi:10.1093/ofid/ofaa075 32195291 PMC7075487

[52] Cendejas-Bueno E, Falces-Romero I, Laplaza-González M, Escosa-García L, Schuffelmann-Gutierrez C, Romero-Gómez MP, Verdú-Sánchez C, Calderón-Llopis B, Amores-Hernández I, Pemán J, Gómez-Zamora A, Río-García M, Menéndez-Suso JJ, Rodríguez-Álvarez D, Durán-Lorenzo I, Pérez-Acosta E, Rubio MR, Álvarez-Rojas E, Martínez-Romillo PD, Goded-Rambaud F, de Lorenzo AG, Maseda E, Mingorance J, de la Oliva P, García-Rodríguez J. 2021. Candidemia diagnosis with T2 nuclear magnetic resonance in a PICU: a new approach. Pediatr Crit Care Med 22:e109–e114. doi:10.1097/PCC.0000000000002548 33044414

[53] Lucignano B, Cento V, Agosta M, Ambrogi F, Albitar-Nehme S, Mancinelli L, Mattana G, Onori M, Galaverna F, Di Chiara L, Fragasso T, Bianchi R, Tortora F, Auriti C, Dotta A, Cecchetti C, Perdichizzi S, Raponi M, Onetti Muda A, Nerini Molteni S, Villani A, Locatelli F, Perno CF, Bernaschi P. 2022. Effective rapid diagnosis of bacterial and fungal bloodstream infections by T2 magnetic resonance technology in the pediatric population. J Clin Microbiol 60:e0029222. doi:10.1128/jcm.00292-22 36069557 PMC9580347

[54] Seitz T, Holbik J, Hind J, Gibas G, Karolyi M, Pawelka E, Traugott M, Wenisch C, Zoufaly A. 2022. Rapid detection of bacterial and fungal pathogens using the T2MR versus blood culture in patients with severe COVID-19. Microbiol Spectr 10:e0014022. doi:10.1128/spectrum.00140-22 35695564 PMC9241933

